# Parry response to *Dialectical Anthropology* forum on *Classes of Labour*

**DOI:** 10.1007/s10624-023-09692-x

**Published:** 2023-05-02

**Authors:** Jonathan Parry

**Affiliations:** Department of Anthropology, London School of Economics, London, UK

## Abstract

The paragraphs that follow respond to some of the criticisms and comments that the contributors to this forum have made on my book. Many of these revolve around the central issue of social class and around my analysis of the manual blue-collar workforce of the central Indian steel town of Bhilai as sharply divided between two ‘classes of labour’ with separate and sometimes antagonistic interests. Some earlier commentaries on this argument had been sceptical, and many of the observations made here invoke much the same issues. In the first part of this response, I attempt to summarize my central argument about the class structure, the main criticisms of it, and my earlier attempts to answer these. The second part responds directly to the observations and comments made by those who have so generously participated in the present discussion.

I am flattered by the kind comments that the colleagues who have contributed to this forum make about *Classes of Labour*. When they sometimes reveal that I cannot have made myself clear, it is however hard to believe them merited. In the face of such generous critics, I can hardly complain, though I hope I may be forgiven for briefly re-stating an argument I have re-stated before. That was in response to a firm rebuttal of one of the central propositions of the book by that doyen of Indian labour studies, Jan Breman (2021). The distinguished historian, Ravi Ahuja (2019), had earlier raised a similar concern. Though my answer to them had, I deluded myself, clarified my position, even defended it convincingly (Parry 2021), echoes of their critique recur in several observations made here. While I cannot explain myself at all without re-treading some of the same ground, it would plainly be pointless to replay the whole record again. Interested readers can refer to my earlier, more detailed response.

What the book set out to show is how the non-managerial workforce of the central Indian industrial town of Bhilai — which grew up around a gargantuan public sector steel plant built with Soviet collaboration as part of the Second Five-Year Plan (1956–1961) — is divided between two distinct ‘classes of labour’. Though Bhilai’s labour landscape is lined with contours and the terrain furrowed by inclines and undulations, there is one really deep rift that sets one segment of the manual workforce apart from the rest. Those on either side of this fissure are separate social classes in the classic Weberian sense: they are sharply distinguished from each other by the lifestyles and the life chances of those who belong to them; mobility *between* them has become highly restricted, while mobility between different occupational positions *within* them — both during the course of a single lifetime and across generations — is comparatively frequent and easy. Their material circumstances and consumption standards are strikingly divergent; their interests are distinct and sometimes in conflict, and there is in some circumstances a relationship of exploitation between them. Their engagement and experience with unions and labour politics are not at all the same; the attitudes, beliefs and values that are characteristic of their members are in important ways different; and there are significant variations between them in terms of household composition, the socialization of children, the stability of marriage, the social and emotional investment in the conjugal bond and in the propensity to suicide.

The crucial fault line, the salience of which is clearly recognized on all sides, is between those who ‘have’ *naukri* (which is spoken of as a possession) and those ‘do’ *kam*. *Naukri* is a ‘service’ position; it is secure employment protected by labour law and remunerated by a regular wage that supports a ‘decent’ standard of living. Its pre-eminent (and statistically predominant) form is *sarkari naukri*, a regular government post (even one that is menial and unskilled). In opposition to it, *kam* — otherwise ‘work’ in general — refers to an irregular and untenured job, paradigmatically in the informal sector and paid by a daily wage. The distinction between the two types of workers is between, on the one hand, a comfortably off labour elite with jobs that are so secure that they all but constitute a property right and on the other hand those these *naukri-vale* (‘service-wallahs’) differentiate from themselves by appropriating for them the English label ‘labour class’. Most ‘labour class’ people work in jobs that are chronically insecure and pitifully paid; most must live for the day and close to the brink of destitution — into which a death, or a serious illness or some other domestic disaster constantly threatens to precipitate them and their household members. In Bhilai, the possession of a secure salaried post is at least as important a marker of class difference as the ownership of land or of other forms of tangible property.

My analysis is unblushingly revisionist. It harks back to an essentially dichotomous portrayal of India’s workforce, seen as bifurcated by the legal and bureaucratic distinction between a regulated ‘organized’ sector and an unregulated ‘unorganized’ one, a distinction that closely approximates that between the ‘formal’ and ‘informal’ economies. The obvious proviso, however, is that many employed in the organized/formal sector are casual workers who do not receive anything like the same pay or the same range of benefits and who do not enjoy the same rights of union representation or of legal job security as regular company workers with permanent positions. Their situation is far closer to that of workers in the unorganized/informal sector. The fundamental rift in the ranks of labour, as this would suggest, might therefore be identified as that between organized sector company employees with permanent positions and all the rest (including the contract labour that works alongside these regulars).

Though the emic distinction between *naukri* and *kam* that my analysis emphasizes as more socially and politically salient significantly overlaps with this etic dichotomy between organized and unorganized, it is not entirely congruent with it. While in local parlance all who have regular *government* jobs have *naukri*, only the small minority who have permanent company posts in a handful of the largest, most modern and best paying *private* factories in the organized sector are described in this way. The crux is that in the private sector, it is only they who have meaningful job security. In terms of the distinctions revealed by my informants’ categories, and I contend of down-to-earth social reality, the critical class divide is thus between, on the one hand, those with *sarkari naukri* plus a small elite of private sector workers with regular posts — a privileged minority even within the privileged minority of private enterprises in which they are employed. On the other side of the divide is the rest of the workforce, including the large majority of those who work in privately owned organized sector factories but are employed in them as untenured contract labour precisely so as to evade the labour laws.

In earlier writings, Breman had himself signed up to a dualistic view of the Indian labour scene, but had soon recanted, to insist that the hierarchy of labour is made up of multiple gradations (Breman 1994: chapter 1 [1976]). At much the same time, much the same trajectory was followed by other prominent commentators on Indian labour, like Mark Holmström and John Harriss (Parry 2020: chapter 2:3). Though nobody ever said so, what was being surreptitiously abandoned was a conventional class analysis, a critical hallmark of which is that it seeks to identify the main structural faults that run through the social order, faults to which the most serious disturbances on the political landscape can be traced and which divide society into a small number of distinct blocks. Offered in its place was a social stratification model which portrays it in terms of multiple ranks on a continuum and multiple criteria of ranking that result in discrepancies between a person’s rank on different scales of evaluation. *Classes of labour* set out to suggest that, at least for the Bhilai context with which I was centrally concerned, there is much to be said for resurrecting a class perspective that focuses on the fundamental structural faults that run through local society. The essence of the Breman-Ahuja critique was to reject the validity of the dichotomous picture of the labour force that results in favour of one that better brings out its more complex layering.

It should not, however, be inferred that my analysis represented either Bhilai’s labour elite or its ‘labour class’ as homogeneous and undifferentiated categories. To the contrary, it went out of its way to document at least some of the distinctions and inequalities that undeniably exist on each side of that divide. It showed, for example, how certain occupational niches had to a notable extent become the preserves of a particular caste, regional group and/or confessional community. It’s a familiar enough phenomenon: in Bhilai, most scrap merchants (*kabadis*) are Muslims from Bihar or UP; men of Carpenter caste from those two states and from West Bengal dominate the pattern shops that make wooden templates for the foundries in its large engineering companies. The incidence of such enclaving is, however, somewhat lower than the comparative literature might suggest and in no instance that I know of did opportunity hoarding of this kind result in an impenetrable monopoly. The broader picture was rather of considerable mobility between all the most common occupational roles *within* each of the two main classes of labour (and especially within the ‘labour class’) and over time increasingly restricted mobility *across* the divide (especially in an upwards direction). While it is an incontestable fact that the hierarchy of labour is fractured by numerous fine gradations and distinctions, the crucial point is that these are but surface cracks as compared to the deep structural rupture that is ultimately the product of state policies and legislation and that divides Bhilai’s manual labour force between two distinct, separate and in some respects opposed *social classes*. True, there are also low hills and shallow valleys, but what really dominates the landscape is one very deep canyon. Socially, politically and economically, what is most significant about Bhilai’s workforce is not the plethora of fine *gradations* within it; it is its bifurcation into two distinct *classes* of labour — *naukri-vale* and ‘labour class’.

But even if we accept the validity of that picture for Bhilai, how applicable is it to the Indian workforce more broadly? And, as Breman also asks, even if once it were plausible, how can it possibly remain so? Narendra Modi was elected Prime Minister in 2014, the year in which I concluded my fieldwork. His BJP-led administration has since significantly advanced an already ongoing government agenda of hollowing out the organized sector, undermining union power and dismantling the protections that Nehruvian era labour legislation had provided to workers. The organized sector, that is, has been subjected to an accelerated process of informalization, as the result of which the erstwhile advantages of the labour elite have been greatly eroded, the gap between the two kinds of worker greatly reduced and any sharp divide between them that may once have existed has been considerably blurred. In sum, my analysis must address three pointed challenges: (i) is it valid even for Bhilai? (ii) Does it apply more broadly? And (iii) if ever it did, does it continue to do so today?

Breman would say ‘no’, ‘no’ and ‘no’ — even if he addresses the first of these questions only obliquely. Rather than interrogate any of the evidence with which I supported (some would say overloaded) my analysis, he mainly casts doubt on it by addressing himself to the second question. My Bhilai account is suspect because it does not ring true for other industrial contexts of which he has first-hand experience. That might be reasonable were it not that I was never claiming that the situation I described for Bhilai is generalizable across the board. To the contrary, I explicitly acknowledged that it was probably an extreme (if not perhaps unique) instantiation of a pattern that is the product of very general sociological *processes* that have to do with class formation and that operate both within and beyond South Asia. The generality, that is, belongs to the level of processes and not at the level of the concrete manifestations in which these processes result. It is somewhat akin to Edmund Leach’s ‘topological’ take on seemingly quite different kinship ideologies as permutations on a common structure, to his famous rubber sheet on which some geometrical figure is drawn. By stretching the sheet this way and that, you produce endless transformations of the design that look very different though they remain in some sense the same figure all along (Leach 1961: chapter 1). The difference, however, is that it was not only the different structural variants that interested me but more especially the social processes that generate them — the forces that, as it were, pull at the sheet and alter the resulting pattern.

As I would see it, the underlying template — the ‘deep structure’ so to speak — was laid down by the Nehruvian state which through its industrial policies and labour legislation created (or at least promoted) a fundamental division in the workforce in terms of pay, conditions and security between those whose employment was significantly protected by the law and those for whom that legislation was largely irrelevant. The proclaimed aspiration was that the former would provide a model for the latter, who would somehow be able to ratchet themselves up into a comparable position. That unsurprisingly never happened, and over time, the gap widened and became increasingly entrenched. The essence of my argument was that the extent to which that took place — to which the two kinds of workers were crystallized into two distinct classes of labour — was contingent on the interplay of a limited number of key variables that combine to produce what Anthony Giddens (1975) somewhat ponderously labelled ‘class structuration’, the process by which groups which share the same economic circumstances constitute themselves as *social classes* with a distinctive sense of their own separate interests and identities. It is essentially a matter of class closure, of the extent to which the group insulates itself in its interactions with others and develops a sense of its difference from them. Structuration, that is, is a matter of degree, and it is advanced to the extent that social mobility between classes is low; that interactions between members of different classes in the workplace, in the neighbourhoods in which people live and in associational life are restricted; that bonds of kinship, marriage and friendship rarely cross class boundaries and the extent to which members of different classes differ in their consumption behaviour, in their political interests and ideals and in their characteristic attitudes and values.

My proposition was that in the case of Bhilai, a series of contingent circumstances of this kind had produced a perhaps unusually high degree of class closure, of structuration, amongst the labour elite; and in response to Breman’s objection that in the case of Ahmedabad — his chosen counter-example — the industrial workforce has a very different character, I suggested on the basis of some of his own ethnography that that is just what my structuration hypothesis would lead one to expect. In Ahmedabad, the processes of structuration had been comparatively attenuated, and the labour elite had never separated itself off as a distinct class in the way that it had in Bhilai (Breman 2004; Parry 2021). *Caste* loyalties had continued to stunt emergent *class* identities; caste played a more prominent role both on the factory shop floor and in the residential neighbourhood, and caste inhibited class closure by providing a powerful alternative focus for identification and association. Characteristically, work groups in the Bhilai Steel Plant were, by contrast, extremely heterogeneous in terms of caste, religion and regional ethnicity, and that was also true of the streets and apartment blocks these workers inhabited in the company township and of the new middle class housing colonies in which many of them had built homes alongside the homes of white-collar workers and junior managers. From these residential spaces, however, ‘labour class’ people were almost completely excluded. In these two very different industrial settings, at both ‘work’ and in ‘life’, workers are aggregated or disaggregated from each other on the basis of class, caste and regional ethnicity in quite different ways. Class structuration has solidified to a far greater extent in the one case than the other, and its history allows us to get a good way towards explaining how that variation comes about.

What is probably equally crucial to understanding that difference today is that the Ahmedabad workers of whom Breman writes were predominantly employed in private sector industry, while Bhilai grew up around a public sector enterprise, employment in which has been the very epitome of *naukri*, its all but exemplary instance. Regarding the proposition that, whatever the past, for contemporary neo-liberal India, it is no longer remotely plausible to postulate such a sharp divide between the two classes of labour, there can be no denying that the state has in recent years sought to cheapen the labour of organized sector workers and to make them more malleable by undermining their security. What is less clear is the extent to which it has managed to do so. The indications are that its success has been patchy (Parry 2021). With some enthusiastic collusion from the employers, its objectives seem to have been easier to accomplish in private sector industry than in the public sector, in which the workforce has generally proved less vulnerable to wage-cuts, lay-offs and enforced redundancy. Undeniably, the size of the *regular* workforce — the workforce with *sarkari naukri* — has been considerably pared in many state enterprises (as it has in the Bhilai Steel Plant). This has, however, been largely accomplished by natural attrition and voluntary retirement rather than by involuntary severance. Undoubtedly, such jobs are now harder to come by, but — by contrast with private sector industry — there is little evidence that across the board those who are actually in them are appreciably less secure than they were or that there has been any systematic intensification of their labour or degradation of their working conditions. Rather to the contrary in the case of Bhilai, the nastiest and most arduous of the tasks formerly undertaken by regular employees have been re-assigned to highly precarious contract workers who are paid a comparative pittance for performing them (Parry 2020: chapter 6:3-5). And three decades of neo-liberal reforms notwithstanding, the evidence is that public sector wages have held up surprisingly well, and that in terms of pay, perqs and benefits, those who have *sarkari naukri* have comfortably managed to maintain their considerable advantage over workers of other kinds (ibid, chapter 5:9; cf. Nagaraj 2017). For those with government posts, it is not perhaps quite so clear after all that the gap between those with *naukri* and *kam* has been greatly eroded.

Nor could anybody who witnessed the appalling destitution that resulted from India’s COVID-19 lockdown just after *Classes of Labour* had been published seriously doubt the continued significance of that gap. It was overwhelmingly ‘labour class’ people who were brutally and ignominiously abjected from their workshops and factories, left entirely without means and forced to plod hundreds of hungry miles home to their villages of origin. Meanwhile, those with *naukri* could barricade themselves in and sit out the siege supported by wages that continued to flow into their bank accounts and by the credit they could still count on at their corner *kirana dukhan* (provision store). And if that contrast is not persuasive enough, consider the long trail of incidents that vividly testify to the premium that countless numbers of India’s ‘common people’ place on *sarkari naukri* as the acme of their ambitions for themselves or their children. Witness the 2013 Madhya Pradesh ‘Vyapam scam’, which involved fixing exam results for recruitment to government jobs and entry to medical colleges, entailed bribery and corruption on a truly spectacular scale and supposedly triggered more than 40 murders and suicides (ibid. p. 68). Witness the violent demonstrations that swept through fifteen Indian states in mid-2022 and that involved torching trains, disrupting transport links and destroying public property and calls for a country-wide strike in protest at the government’s Agnipath scheme. That proposed restricting all recruitment to non-commissioned ranks in the armed forces to an initial 4-year term, after which only 25 per cent would be reappointed, in effect completely removing the security that government employment has always conferred and for which it is so hugely valued. Or consider the spectacular increment to their support that politicians can achieve with pre-election promises to expand the state bureaucracy and create some phantasmagorical number of new government posts (Parry 2021). Were it the case that the gap between *naukri* and *kam* has dwindled into insignificance, to account for such reactions it would be necessary to suppose that millions of ordinary Indians live in a fool’s paradise in which they obsessively chase shadows, obsessively snatch at now empty husks. The simpler and more plausible hypothesis is that their preoccupation with *naukri* stems from the hard-headed recognition that it is still a serious long-term asset and is quite different from *kam*.

But why does the distinction persist? And why should public sector *naukri* be more resilient to neo-liberal winds? A large part of the answer is obvious: it is politics. That would include the fact that private capital has bankrolled the electoral success of the BJP, and its immediate preoccupation is with reducing the cost and the bargaining power of the labour it employs in its own enterprises. For the paymasters, that is, the priority is the price of *private sector* labour and its amenability to discipline. On the other side of the coin, significant segments of the public sector workforce are more formidable opponents who are employed in ‘commanding heights’ type industries (like coal, steel and the railways). At least potentially, that gives them serious political muscle. Government employees are, moreover, a vote bank of real significance; and without the collusion and cooperation of lower-tier government bureaucrats, the everyday state could never function. As Nagaraj (2017) observes, no political party can afford to antagonize them, not least because they can subtly influence electoral outcomes and because Indian elections require vast financial resources that are often obtained by corrupt means and for which the bureaucracy is a major conduit. Government jobs are a critical resource in the patronage system, and for that reason, state governments routinely choose to curtail investment in infrastructure and services rather than shrink public sector employment.

***

Readers may puzzle that, instead of directly addressing the comments and criticisms of the contributors to this forum, I have thus far mainly rehearsed a critique of an earlier critique. The justification is that so much of what I have to say in response to the present set of interventions is anticipated by that earlier exchange. The key issue once more is labour dualism — my picture of Bhilai’s manual workforce as split between two opposed classes of labour, the degree to which that kind of differentiation is replicated elsewhere and the implications it might have for the wider polity.

I am grateful to Christian Strümpell, who amongst my reviewers is the only one to date to put the structuration argument at the centre of the discussion, who summarizes it accurately and who sympathetically evaluates its comparative intentions and potential. What Strümpell also helpfully brings out are the crucial implications that, on my analysis, structuration has for the politics of labour and more especially for the difficulties that workers outside the labour elite have in claiming basic rights of citizenship. As his own Rourkela ethnography illustrates even more strikingly, class structuration is crucially contingent on the intersection between class and ethnicity. In both Bhilai and Rourkela, it has advanced over time and counter-intuitively has in some respects even *intensified* as a consequence of neo-liberal reforms (*pace* Breman). Despite his clear recognition of the progressive hardening of the class boundaries between *naukri-vale* and the ‘labour class’, however, Strümpell winds up doubting the long-term durability of the labour elite as a distinct class or stratum. The issue is its reproduction. Today, there is very little *naukri* for the boys, many of whom are doomed to slide down the ladder. *Naukri-vale* are a dwindling breed, facing possible extinction. Agreed, that prognostication seems reasonably plausible, though without a crystal ball it is hard to be confident. Its obvious implication is that I am describing a rapidly disappearing world. Even if *naukri* and *kam* have yet to blend into each other, they soon enough will. Breman will then have been vindicated. ‘Plausible’ is not however probable, let alone inevitable. As I tried to suggest a couple of paragraphs back, a variety of powerful political imperatives — at least for the present —- provide the employment structure with a significant degree of prophylactic inertia. *Naukri* is highly resistant to change and hard to abolish. Entrenched interests are at stake. That makes eventual outcomes very uncertain.

Though less explicitly, that tension is also woven through Ashima Sood’s comment, which draws attention to ‘the increasing momentum of dualism in the neoliberal steel town’ while at the same time stressing the pressure ‘to partial divestment and privatization’ to which these public sector steel plants are exposed and the likely effects that has on employment security. As just suggested, however, there are countervailing forces that mitigate the pressure and thereby buttress the dualism. In a different direction, Sood’s contribution helpfully contextualizes my Bhilai ethnography in relation to the burgeoning literature on the Indian middle class(es) to bring out the sheer serendipity that was often involved in getting Bhilai Steel Plant *naukri* in the first place, the canny strategizing it often took to pass it on to the next generation, the ‘neighbourhood effects’ that mediated this process and the deep insecurities provoked by problems of class reproduction and the suicidal anxieties these create in the labour elite. *Classes of Labour* shows that, in terms of the size of the purse and in terms of their ‘status situation’ (and by contrast with private sector industry), the gap between blue and white-collar workers in the Bhilai Steel Plant has become considerably narrower and easier to bridge than the gap between regular BSP manual workers with *naukri* and temporary BSP contract workers without.

The *naukri/kam* distinction became ingrained as the consequence of state policies and regulations integral to a Nehruvian nation building project that had the probably well-intentioned, but ultimately half-baked, objective of fostering a ‘modern’ industrial working class for a new ‘socialist’ India. ‘Half-baked’ because the protections afforded were conferred only on a very small segment of the manual workforce. The rest were left in the cold, with inevitable consequences that cut the ground from under the supposed socialist goal. In Nepal, as Mallika Shakya points out, the politics were very different and a labour aristocracy of the kind we find in India did not develop. What we badly need, she suggests, is a comparative analysis for different South Asian countries of the relationship between the way in which labour is structured and organized on the one hand and state building trajectories on the other. That’s an important and interesting project but not one that I had either the space or the competence to broach in my book.

Naturally, it is immensely buoying that a friend and former student should describe *Classes of labour* as a ‘masterpiece’ — in, of course, the historical sense of a piece of craft work submitted to a guild as evidence of competence by a person aspiring to be recognized as a fully qualified craftsman. At the same time, it is somewhat deflating that in almost the same breath Andrew Sanchez should so summarily dismiss its central argument as without merit. I may have made a very fine chair, he in effect says, but nobody should trust their weight to it. Some competence, one might think, when the craftsman’s creation won’t even stand up. Its first major defect is that while the emphasis I place on the *naukri/kam* distinction may be valid for Bhilai, it is doubtful that as a conceptual framework it illuminates class more broadly. Its second fatal flaw is that that distinction is too malleable and ephemeral to be of real significance. Under the conditions of contemporary capitalism, the line between the securely and the precariously employed is (as Breman claimed) being rapidly effaced.

Regarding the first of these claims, and I repeat myself, the qualification that I have consistently attempted to register is that I do not assert that the clear class divide between *naukri-vale* and *kam karne-vale* (‘those who work’) that obtains in Bhilai emerges with the same sharpness throughout industrial India. Its foundations have been laid by the laws and policies of the state, and at least as a potential — and even if in some more inchoate or vestigial form — it therefore exists across the country. Its emergence in the highly concretized variant that I have described for Bhilai is the result of the interplay of several key parameters that have crystallized the sense that these groups have of their separate and opposed identities. From the data I have instanced, however, and though perhaps in weaker form, the importance of some such a distinction for many millions of ordinary Indians beyond Bhilai is nevertheless plain. It seems curious that Sanchez does not find it necessary to so much as mention, let alone critically engage with, any of that empirical evidence. It would be instructive to learn, for example, how *he* would explain the tragically sharp divisions that emerged within the workforce at the time of the COVID-19 lockdown? How would *he* understand the Vyapam scam or account for the electoral dividend that politicians derive from promising to expand the number of government posts? And what does he read from the fact that *sarkari naukri* has remained so comparatively remunerative?

As to his second point — about the permeability under neoliberal conditions of the boundary between secure and precarious employment — it has been a bone of contention between us before. In the context of his own remarkably revealing data on the Tata Motors workforce in the Indian city of Jamshedpur, Sanchez (2018) earlier emphasized the increasing precarity of a regular workforce that was once a byword for job security. Their conditions of employment were being eroded and the differentiation between them and temporary workers had in important respects been reduced. On his own evidence, however, what struck me was how salient that distinction remained (Sanchez 2016: chapter 6; Parry 2018). What his ethnography unequivocally showed was that regular workers could with impunity and calm insouciance get away with blatant malingering, bloody-mindedness, even insubordination (which was why management employed as few of them as possible). No temporary worker who wanted to stay in his job could possibly behave in that way. And Sanchez makes it clear that one of the main reasons that these temporary workers submit to much harsher conditions under a stricter disciplinary regime for much poorer pay, and work so much harder, is in the hope that one day they will themselves be one of the lucky few to be upgraded to a permanent job and that then they too will be able to skive. How, one might wonder, is all that consistent with the proposition that there is no longer much difference between the two employment statuses?

Geert De Neve identifies three broad problem areas in my analysis that I find particularly challenging and that I am not confident I can adequately address. The first stays on familiar terrain. It again relates to the generalizability of my Bhilai class analysis and my disagreement with Breman. There is no call, De Neve emolliently suggests, for dispute. It is entirely possible that both of us are right for the particular context about which we principally write. While I do not accept his charge of having played down the specificity of my own ethnography, I agree that there are important differences in industrial organization and in class structure between Bhilai and Ahmedabad. I am therefore happy to assent to the proposition that my dichotomous two-block analysis of Bhilai’s classes of labour cannot be simply transposed to Breman’s Gujarat setting, and I agree that in that instance it may make sense to foreground the multiplicity of gradations that punctuates the hierarchy of labour. Indeed, I had thought that that is more or less what I have said all along. That said, I do not however wish to retract my claim in *Classes of Labour* (pp. 53–4) that there are solid empirical grounds for suspecting that, in his anxiety to dissociate himself from his own earlier more dichotomous analysis, Breman (1994) went too far in downplaying the extent to which Ahmedabad’s labour elite is set apart from workers of other kinds. Once again, it does not seem unreasonable to suggest that we discuss the data; whether it supports that charge and whether it does or does not show that in Ahmedabad also workers with *naukri* are set significantly apart and may even belong in a separate class of their own. Regardless of the degree of their separation, however, I have no problem in conceding that there are significant differences in the configuration of classes between the two contexts.

Perhaps trickier is how Tiruppur fits in. That is the South Indian textile manufacturing town in which De Neve himself has done much of his fieldwork. It is a very different type of industrial conglomeration from either of these, and it immediately invites questions about how my class analysis for Bhilai might apply to places where ‘the landscape of labour is not organized around a dominating (state) enterprise’ such as BSP. What light can that analysis shed on contexts like this in which the economy is overwhelmingly informal and in which almost all employment is provided by small units with pay and conditions that vary considerably but nobody has the kind of security conferred by *naukri*? Might not a picture of multiple breaks and a ladder-like hierarchy be more apposite? And is that kind of informal sector scenario not considerably more common than the one I describe for the public sector steel towns?

This issue of typicality may not be easy to resolve. Arguably, Bhilai and Tiruppur might appear to be located at opposite poles on a continuum along which many hundreds of intermediate cases are ranged. It would be unwise to take either as the norm. It is worth remembering, however, that Tiruppur cannot be so very different. It too must have its cohorts of employees with *sarkari naukri*. It has one of the busiest stations in the Southern Railway Zone and presumably a considerable number of workers for the nationalized railway system. It has several branches of the State Bank of India, a collectorate and courts, a number of police stations and government schools, and many other state institutions — all contributing to a considerable army of lower-level state functionaries with pay, perqs, and conditions comparable to workers in BSP. If we are interested in the class structure and in class relations, we surely cannot think of the workforce too narrowly, as confined to those employed in industrial workshops and factories. And as soon as we expand the lens, some kind of dualism comes into view between those who have something like *naukri* and those who do not. Tiruppur and Bhilai are not two entirely separate worlds after all.

Further, and while I would have to concede that my two-block analysis of Bhilia’s classes of labour may shed limited light on Tiruppur complexities, I see no reason why an analysis in terms of the processes that have generated that class configuration — an analysis, that is, in terms of structuration — should not be equally applicable to those complexities and should not provide a comparative frame that would illuminate both contexts. Across the board, such an approach might provide us with a coherent set of research questions which would be relatively straightforward to operationalize (about rates of mobility between different strata; about the incidence of inter-marriage and the prevalence of kinship, friendship, and neighbourhood ties between them; and about all the other obvious indices of class closure). Across the board, it would enable us to identify breaks on the hierarchy of labour, locate their source (the variables that give rise to them), form some estimate of their significance and depth, and thus assess whether they constitute a major *class* divide within the ranks of manual labour or merely mark one of its many gradations. Admittedly, a series of case studies along such lines might be hard to aggregate into a composite picture of *the* class structure of the Indian industrial workforce as a whole — as if such a thing were even possible. Certainly, in the present state of our knowledge, it might be more than sufficiently ambitious to aim at identifying the dominant variants, at being able to say something about their distribution, and perhaps even about the conditions under which they morph into each other. De Neve wants an agenda. I believe that my book has already proposed that processes of structuration provide a promising place to start.

His second ‘reflection’ is sparked by the brief case history of a successful construction contractor I call Kedar Nath (Classes, p. 366). It is a story of upward mobility, of a man of ex-untouchable caste from rural Bihar who as a youngster had worked as a building site coolie in various parts of north India, had learned the skills of a mason, and had started to take on housebuilding contracts after he came to Bhilai. When I first met him, he was employing a workforce of 50–60, and as my fieldwork drew to a close, he had just won what promised to be a highly lucrative contract building multiple social housing units for a government scheme administered by the municipality. By then, he had invested in a truck and cement mixers, had bought fields in a peri-urban village adjacent to Bhilai, and built a home in one of its up-market housing colonies. To all appearances, he had made it into the middle class(es). But how, asks De Neve, did his social position compare with that of BSP workers whose *naukri* conferred that status? And does not his example suggest that there may be a variety of different pathways up the class ladder, and that my account may be somewhat misleading in so heavily privileging the BSP route?

As it happens, I remain in occasional telephone contact with Kedar Nath and can add a few details that update his story. De Neve’s inferences about situations like his are sound. Over recent years, he has almost certainly made an income that dwarfs that of even a BSP worker at the top of the pay scale, even if it was not so dependable. It was ever thus. From the outset, he had several false starts as a contractor, got landed with bad debts, and had to return to working as a mason before beginning again. ‘See here, Sir,’ he explained when last we spoke, ‘being a contractor is like this: you get it (the contract, the sum agreed) and you are a millionaire; if you don’t you are the heir to ashes’. He was speaking of what happened at the end of his big-break housing board contract. His bills were disputed, and Rs 10 million was withheld (roughly €112.6K). He had to sell vehicles and land to discharge his debts, and it was not until a new commissioner was appointed that a settlement was agreed and he was able to recuperate some of it. And then last summer (2022), his wife died after a protracted illness during which they incurred crippling medical bills and he was unable to properly attend to his business. In that situation, the wife of a BSP worker would have been treated free in what is still the best hospital in the region, and he would have been granted compassionate leave to look after her. Kedar Nath’s three children have been privately educated in an English-medium school (though not one of the more prestigious ones), and now three grandchildren are at another run by a Kerala Christian foundation. His daughter is married to a railway worker posted locally, and the eldest boy, qualified as an electrical engineer, briefly ran contracts for the State Electricity Board but was unable to make a go of it and now works for a Tata Company subsidiary which has maintenance contracts with the Bhilai Steel Plant. His younger brother is also a university graduate, but Kedar Nath despairs of him. He is feckless and indolent, and his father does not know what he does all day. He and his sons and their families live jointly; and their house and its ambience are distinctly middle class.

De Neve is right. There are other routes up the ladder and becoming a contractor in the building industry is one. Tara provides a second example (*Classes*, p. 138). Initially, he too made money in construction and later a good deal more through real estate. While his brothers live mainly in penury, his household is seriously rich, and his son is the CEO of a private management college that charges hefty fees for providing professional training. Statistically, however, I judge that Ankalaha is much more typical (Ibid, p. 366). He too is of Dalit caste and followed the same path as Kedar Nath: coolie ➔ mason ➔ contractor. At the peak of his career, he was employing an even larger workforce, though by the time I first met him he was operating on a much reduced scale (a dozen workers at most), and when I wound up my fieldwork his circumstances were seriously straightened. He was ageing, had been ill for some months, and unable to work, his wife’s brother’s son who had been his righthand man had unexpectedly died, his son was a wastrel who never worked, and his household of six was getting by on the wage of an unmarried daughter employed in a just opened branch of Domino’s Pizza. In his line of business, it is easy to slip through the cracks. A *naukri-vala* would be unlikely to slide down the ladder with comparable velocity.

Apart from a BSP post, or making it as a contractor of some kind, there are other options, though historically all have been chancier and unlikelier than the first of these routes up the ladder. Especially in real estate, operating as a broker or commission agent — generically known as *dalali* — can conjure rapid fortunes for those lucky or ruthless enough (Ibid. chapter 4.3), and I knew a couple of plausible men in early middle age who did very well marketing life insurance policies. One now prosperous shopkeeper I remember as a very young lad selling second-hand schoolbooks on the roadside. His charming but lackadaisical father was a *pheri-vala* who went door to door buying up paper and cardboard for pennies to recycle (Ibid. p. 390). And I remember the father deliberating about when he should initiate his son into dealing in a particularly profitable line — the pornographic magazines that would sometimes come his way on his rounds. Now that boy runs the biggest second-hand book business in Bhilai and last year purchased a prime site shop for Rs 9 million.

There is, as De Neve’s line of questioning exposes, much more to be said about contracting, and about other entrepreneurial activities, in relation to class and to class mobility. This was an issue that Suravee Nayak (2021) also touched on in a thoughtful review of the book, and to it she adds valid criticism of my treatment of the ‘buffer-zone’ that I identified as lying between the two principal classes of labour. This I characterized as an amorphous catch-all category of occupational positions without a unifying sense of identity, a category into which the sons of the labour elite are most likely to fall if they fail to get *naukri*, and the children of the ‘labour class’ are most likely to rise if they are able to rise at all. Included in it would be shop assistants and shopkeepers with businesses of varying scale; workshop owners of different kinds, owners of small taxi and transport businesses, land *dalals*, labour recruiters, and contractors of many sorts. It is clearly a significant, if nebulous, social space, and Nayak rightly complains that I say too little about it. More bluntly put, she might justly have said that my conceptualization of it is inadequately developed and my ethnography of it is comparatively thin.

De Neve’s third problem area has to do with the political implications of the dualism I find in the way in which the workforce is structured. The labour elite and the ‘labour class’ are quite separate; their interests are different and sometimes opposed. The labour elite has industrial muscle; but from the outset, it also had privileges in terms of pay and of welfare provisions that were given them gratis from on high as part of Nehru’s nation building project and that never had to be fought for. It had no need of ‘labour class’ allies, saw its interests as different from theirs, and had little incentive to become champions for the citizenship rights of the generality of working people. It never saw itself, nor became, ‘the vanguard of *the* proletariat’. If there was such a thing, these workers had no reason to suppose that they were part of it. Why would they use their power to claim rights on behalf of a collectivity to which they did not belong? To a significant degree as a consequence of that rent in the ranks of labour, I suggested, the Indian state has been able to get away with treating most of the labouring poor, the informal sector ‘labour class’, as denizens rather than citizens. It is T.H. Marshall (1992 [1950]) in reverse. Rather than the equal claims of citizenship mitigating the inequalities of class as up to a point happened in western Europe, what is most visible here is the other side of the coin, the way in which class inequalities have undermined the equality embedded in the rights of citizenship.

De Neve’s reaction here is redolent of Rina Agarwala’s (2013) rather more optimistic diagnosis when she argues that in recent years organizations championing the cause of informal sector labour have made significant gains by pursuing welfare claims against the *state* as *citizens* rather than by extracting concessions from the *employers* as *workers*. De Neve asks if campaigns like Right to Food and Right to Work do not hold out hope of more progressive developments through a reassertion of broad citizenship claims against their subversion by the class forces I identify. It is through such campaigns, that is, that the structural fissure by which labour is divided is perhaps to be overcome. We may hope that is so, though it is hard to say that it is. On the long-term impact of such campaigns the jury is out. Much must depend on the power and commitment of the political support that such movements are able to mobilize and sustain. The general picture is undoubtedly patchy. At least with regard to *legal* rights of citizenship, it cannot be claimed that the record of the Indian state, or of the state government of Chhattisgarh in which Bhilai is located, has over the past two decades been at all reassuring. Citizenship rights have not always fared well.

An important strand in the analysis that runs through *Classes of Labour* concerns the intersection between caste and class. I am grateful to Jayaseelan Raj for airing that issue here — even if I do not recognize my position in his spirited critique of it. I probably have myself to blame. The book is too long, life is too short, and Raj has trusted too much to what an earlier reviewer says that I said in a friendly, pithy, and not quite accurate summary of its argument. It is really, as I see it, with Dipankar Gupta (2020) that Raj should be picking his fight, not me.

In the opening paragraphs of the book I announced that:

Although distinctions based on class, caste, gender, religious identity and regional ethnicity retain real significance, and although these various forms of differentiation intersect and overlap in complex ways, my argument will be that, in most everyday interactions, class now trumps caste as the dominant axis of inequality. It is class that most decisively determines life chances and that is ideologically stressed. (*Classes*, p. 3–4)

Gupta enthusiastically endorses this assessment as applicable to India as a whole. Raj follows suit in treating it as a general proposition but disputes its validity. It is not only wrong but reactionary. It is a product of the ‘positionality’ of its proponents, a top-down high caste perspective on Indian society. That is apparently a fact so self-evident that it requires no elaboration — unless for the instruction of a handful of atavistic academic dinosaurs bewildered by the memory of M.N. Srinivas being castigated as a reactionary for the attention he paid to the continuing significance of caste in modern India and for downplaying class (Srinivas 1995). When it comes to our own biases, few of us are greatly gifted with clear-sightedness, and I am certainly chastened to learn that — despite the fact that most of the closest relationships I formed in Bhilai over a 20-year period were with families of ex-untouchable caste — I have wound up as a propagandist for the twice-born. Of the evidence of that I am unclear, though Raj does explain that one corollary of the ‘hasty denial of caste as a central category of identity in India’ is a denial of caste violence and of the discrimination that Dalits experience daily. Apart from scattered references throughout the text, I thought I had said a good deal about that in three substantial sections of chapter 9 on ‘conflict and violence in the neighbourhood’ (pp. 438–45), on ‘caste in the neighbourhood’ (pp. 450–64), and on ‘caste atrocities’ (pp. 464–72). I am genuinely perplexed.

For the record, I have never claimed that caste is being, or has been, superseded by class throughout Indian society and that proposition is at total odds with the whole spirit of my analysis. *In Bhilai*, I intended to show the structuration of *classes* has over time intensified, while the structuration of *castes* has been blunted. The two axes intersect to produce a configuration which is at the same time particular to time and place and the product of general processes. Caste, as I made clear at the outset, is not the same everywhere:

It is a truism that the children of an Anglophone professor in a metropolitan city do not experience caste in the same way as the children of a Bihari peasant. Caste has a different significance and meaning at different levels of the class and occupational hierarchies, as also in large towns and small villages. Rather than searching for macro-level generalisations about the role of caste in contemporary India, it might be more illuminating to focus on the variations and on how to account for them. (Classes, p. 26, emphasis added)

In line with that, I argued that one reason for the relatively attenuated significance of caste in Bhilai has to do with the sheer social and regional heterogeneity of the workforce that built its steel plant and settled in its township, and I argued that — as compared to BSP — the greater prominence of caste on the shop floors of the Bokaro Steel Plant and of the Bangalore telecommunications factory studied by Subramanian is correlated with the much greater dominance of local sons-of-the-soil in their workforces. In the Bangalore case, it is also associated — as Subramanian (2010): 654f suggested — with important differences in the work process and in the social organization of work, differences which mean that the shop floor is not the ‘melting pot’ that it is in Bhilai (*Classes*, pp. 649–51). Compared with Chandavarkar’s historical study of the Bombay workforce (Chandavarkar 1994): 122, which argued that industrialization had in some ways strengthened caste, I argued that very different material conditions in Bhilai had favoured the opposite outcome (Ibid p. 24).

Far more important for me than these contrasts in the salience of caste between different industrial cities, however, are the differences and variations in its significance between different milieux *within* Bhilai and the possible explanations for these variations. So, for example, I endeavoured to show that until rather recently, and largely as a result of the way in which labour was recruited through contractors in order to evade the employment laws, caste had had a visibility and an importance on the shop floors of private sector factories that it did not have in the public sector steel plant. That gradually changed during the course of my fieldwork, and I tried to explain how and why that had happened. Amongst the local Chhattisgarhi population, I was also interested to show how caste has a different significance and valence for Satnamis (the largest Dalit caste in the region) and for the so-called Hindu castes (which are most of the rest). For the former, caste is ‘a mark of Cain’ that is significantly harder to transcend. The stigma of untouchability, and caste atrocities and violence in the countryside, make it more difficult for the Satnami elite to dissociate themselves from their more disadvantaged caste-fellows and to do what *naukri-vale* of ‘Hindu’ caste increasingly do — identify themselves more by their class than by their caste. It was moreover the case that, because of the affirmative action provisions of the state, the class position of this Dalit elite was often an artefact of their caste. Without job reservations they would not have become *naukri-vale*. That notwithstanding, however, it remained in my judgement the case that *class* was for the children of the Satnami elite the most crucial determinant of life chances — a judgement backed up by numerous family histories. And what my ethnography also suggested is that the role caste plays in daily life is significantly inflected by place of residence. In telling ways, inter-caste interactions differ depending on whether one lives in the BSP township, in one of the numerous new middle class housing colonies, in an old Chhattisgarh village now swallowed up by the town, or in a newly encroached slum squatter settlement. Sufficient said, I hope, to establish that it is something of a simplification to reduce my discussion to the claim that caste in contemporary India is on the way out.

To end on a more consensual note, however, Raj and I can perhaps agree on one transparent proposition: positionality may often be blinding.
